# FGF23 promotes proliferation, migration and invasion by regulating miR-340-5p in osteosarcoma

**DOI:** 10.1186/s13018-022-03483-w

**Published:** 2023-01-05

**Authors:** Lun Fang, Zhongzhe Li, Beilei Yu, Lu Zhou

**Affiliations:** 1 Institute of Sports Medicine, Shandong First Medical University and Shandong Academy Medical Sciences, 619 Changcheng Road, Taian, 271016 Shandong People’s Republic of China; 2 Clinical Center for Sports Medicine and Rehabilitation, The Affiliated Hospital of Shandong First Medical University, 706 Taishan Great Street, Taian, 271000 Shandong People’s Republic of China

**Keywords:** Osteosarcoma, FGF23, miR-340-5p, Proliferation, Migration, Invasion

## Abstract

**Background:**

Increasing evidences have been indicated that FGF23 is associated with the biological behavior of malignant tumors, but its role in osteosarcoma and the specific mechanism need to be elucidated. The purpose of this study is to investigate the effects of FGF23 on the proliferation, migration and invasion of osteosarcoma cells, and the possible molecular mechanisms.

**Methods:**

Western blot was used to detect differences in FGF23 expression in osteosarcoma cells MG-63 and U2-OS and osteoblasts hFOB1.19. FGF23-overexpressing adenoviruses and FGF-silencing plasmids were transfected into osteosarcoma cells, and transfection efficiency was verified using Western blot. MTT and colony formation assays were performed to detect osteosarcoma cell proliferation. Cell cycle was measured by flow cytometry. Scratch assay, holographic imaging cell analyzer Holomonitor ® M4 and transwell were applied to detect cell migration and invasion. Dual-luciferase reporter assay was performed to validate the interaction between FGF23 and miR-340-5p. Changes in miR-340-5p mRNA levels were measured by QRT-PCR.

**Results:**

FGF23 is highly expressed in osteosarcoma cells compared to hFOB1.19. Overexpression of FGF23 significantly promoted the proliferation, migration and invasion of MG-63 and U2-OS cells. MiR-340-5p is a target of FGF23. Transfection of miR-340-5p mimics reversed the promoting effects of FGF23 on proliferation, migration and invasion of MG-63 and U2-OS cells.

**Conclusion:**

FGF23 promotes osteosarcoma cell proliferation, migration and invasion by targeting miR-340-5p gene expression.

## Background

Osteosarcoma (OS) is the most common primary malignant bone tumor, mainly occurring in children and adolescents. It occurs frequently in tubular bones of limbs, resulting in limited joint movement, fractures, and even pulmonary metastasis, with high incidence and poor prognosis. Surgical tumor resection, systemic chemotherapy, and targeted radiotherapy are currently the standard treatment strategies for osteosarcoma [1], and the prognosis and survival rate of patients with non-metastatic osteosarcoma have been significantly improved, but the 5-year survival rate of patients remains very low due to the high aggressive and rapid metastatic nature of OS [2]. Therefore, it is essential to strengthen the study of the molecular mechanism of osteosarcoma.

Fibroblast growth factor 23 (FGF23) is a member of the endocrine FGF subfamily, also including FGF19 and FGF21. Fibroblast growth factor (FGF) signaling networks play important roles in tumorigenesis, tissue repair, and tumorigenesis by regulating cell proliferation, migration, chemotaxis, morphogenesis and angiogenesis [3]. Abnormal FGF signaling can contribute to tumor development by directly driving tumor cell proliferation, invasion and survival as well as supporting tumor angiogenesis [4]. FGF23 is produced by bone cells and is a bone-derived hormone that regulates the metabolism of phosphorus and active vitamin D [5]. Recent studies have found that FGF23 can promote the progression of prostate cancer, and in vitro studies have shown that it can promote the proliferation and migration of prostate cancer cells [6]. Moreover, FGF23 can increase Vegf-a production through autocrine signaling and Egr1 activity, which in turn promotes vascular proliferation in multiple myeloma [7]. To date, however, there is no relevant report on whether FGF23 is able to regulate osteosarcoma growth and migration.

MicroRNA (miRNA) is evolutionarily conserved non-coding single-stranded small molecules that regulate the expression of protein-coding genes by binding the 3 ′ UTR (Untranslation region, UTR) region of the target, resulting in mRNA degradation or translational obstruction [8, 9]. MiRNAs play critical roles in both physiological and pathological conditions, and miRNAs are involved in cancer progression by specifically binding to target genes and regulating the expression of oncogenes or tumor suppressors [10]. Studies have shown that miR-340-5p plays a tumor suppressor effect in the occurrence and development of varieties tumors and has become one of the most promising targets for targeted therapy of tumors [11, 12]. And in OS cells, miR-340-5p expression was down-regulated, inhibiting the proliferation, invasion and drug resistance of osteosarcoma cells [13, 14].

In this study, we examined the difference in the expression of FGF23 in osteoblasts and osteosarcoma and observed the regulation of miR-340-5p target genes by FGF23, so as to clarify the effect of FGF23 on the proliferation and migration of osteosarcoma MG-63 and U2-OS cells, providing new ideas for early diagnosis and gene therapy of osteosarcoma.

## Materials and methods

### Cell culture

Osteoblast cell line hFOB1.19 and OS cell lines MG-63 and U2-OS cells were studied. DMEM medium (Gibco; Lot.8121218, Rockville, MD, USA) containing 10% fetal bovine serum (Biological Industries; Lot.2033119, Kibbutz Beit Haemek, Israel) and 1% penicillin streptomycin (Sigma-Aldrich, St Louis, MO, USA) was used for culture at 37 °C and 5% CO_2_. Subculture is performed when the cell density is confluent to 75–85%.

### Cell transfection and group

Logarithmically growing U2-OS and MG-63 cells were seeded into 6-well plates at a cell density of 2 × 10^5^ per well, and transfected when the cells fused to 30–50%. Cells were infected with FGF23 adenovirus and GFP adenovirus (Hanbio Biotechnology, Lot.xbd-001), and si-FGF23 and si-NC (Hanbio Biotechnology, Lot.KT-001) were transfected into U2-OS and MG-63 cells according to the instructions of the LipofectamineTM 2000 transfection kit (Invitrogen; Thermo Fisher Scientific, Inc., Waltham, MA, USA), while untreated cells were used as the control group, and fresh complete medium was replaced 6 h after transfection. After 24 h of culture, the expression of FGF23 in the cells was detected by Western blot.

Cells were grouped as follows: NC (untreated OS cells, control group), ad-con (control adenovirus), ad-FGF23 (adenovirus transfected with FGF23 overexpression), si-NC (control plasmid), and si-FGF23 (plasmid transfected with FGF23 silencing).

### MTT assay

OS cells were seeded in 96-well plates at a cell density of 2 × 10^3^ cells/well. Transfection was performed for 24 h according to predetermined groups, and five duplicate wells were set for each group. At the end of treatment, 20 μL of 5 mg/mL MTT solution (Biotopped, Beijing, China) was added, and the culture was continued for 4 h. The culture medium in the wells was carefully pipetted, 150 μL of DMSO was added to each well, shaken at room temperature for 10 min in the dark, and the OD value of each well was measured at a wavelength of 490 nm.

### Colony formation assay

U2-OS and MG-63 cells were seeded in 6-well plates at 500 cells/well, cultured for 14 days after transfection, and the medium was changed every 2 days. After the culture, they were fixed in 4% paraformaldehyde (Solarbio; lot.P1110, Beijing, China) for 20 min, added with 0.5% crystal violet solution (Beyotime; lot.C0121, Shanghai, China) and allowed to stain at room temperature for 20 min. Photographs were taken, and the number of colonies formed was counted using ImageJ software. (A colony with a diameter greater than 0.3 mm was counted.)

### Flow cytometry

Cells in each group were collected, adjusted to a cell concentration of 1 × 10^5^ cells/mL, fixed overnight at 4 °C with 75% ethanol solution, cultured for 30 min at room temperature by adding RNase and stained with 50 μg/mL propidium iodide (Solarbio; lot.C0080, Beijing, China) for 30 min at room temperature in the dark.

### Wound-healing assay

Cells in each group were seeded in 6-well plates at a density of 3 × 10^5^ cells/well and cultured until the degree of cell fusion was more than 90%, and the cells were crossed with a 200-μL spear head to artificially create scratches, and the scratches were photographed under the microscope (× 40) after 0 h and 24 h of scratching.

### Transwell

30 μL Matrigel Matrigel diluent (Matrigel: DMEM = 1:5) (Corning; lot.356234, Corning, NY, USA) was evenly spread on the cell inserts, and the inserts were subsequently placed in a 37 °C, 5% CO_2_ incubator until the matrigel completely solidified. The cells in each group were added to DMEM medium without fetal bovine serum, and the diluted cell concentration was 2 × 10^5^ cells/mL. 100 μL of cells were added to the upper layer of the transwell, and 500 μL of DMEM medium containing 10% fetal bovine serum was added to the lower layer and cultured for 18 h. The chamber was removed from the incubator, and the upper cells were gently wiped with a cotton swab, fixed with methanol for 30 min, stained with crystal violet for 10 min, and photographed under a microscope.

### Holomonitor ® M4

Motion trajectories of U2-OS and MG-63 cells were tracked and imaged using a Holomonitor ® M4 microscope [15]. Open the microscope component of the Holomonitor ® M4 system and place it in the CO_2_ incubator overnight to improve the stability of each parameter. Cells were seeded in a matched six-well plate at a density of 2 × 10^5^ cells/well, processed according to predetermined groups, and placed in the instrument. After testing the instrument and device, observe continuously for 24 h, with each time at an interval of 10 min. 24 h later, M4 Studio tracking software 2.6.2 was used to analyze the data.

### Dual-luciferase reporter assay

Target gene prediction using the bioinformatics analysis tools TargetScan database (http://www.targetscan.org/vert_72/) showed that there may be an interaction between FGF23 and miR-340-5p. FGF23 wild type (WT-FGF23) and FGF23 mutant (MUT-FGF23) were constructed and miR-NC and miR-340-5p were transfected to MG-63 cells, respectively. At 48 h of transfection, luciferase activity was measured using a dual-luciferase kit (Promega, Madison, WI, USA) [16].

### QRT-PCR

Total RNA from cells in each group was extracted using the TRIzol kit (BioFlux; cat.no.BSC52M1, Beijing, China), and RNA concentration and purity were measured at a UV spectrophotometer. CDNA was synthesized by reverse transcription following a reverse transcription kit (BioFlux; cat.no.BSB07M2B, Beijing, China), which was used as the template for PCR amplification by fluorescence quantitative kit. Reaction conditions were predenaturation at 95 °C for 2 min, denaturation at 95 °C for 30 s, annealing at 60 °C for 30 s, and extension at 72 °C for 30 s for a total of 40 cycles, with GAPDH and U6 as internal references. PCR primer sequences are listed in Table 1.

**Table 1:** Sequences and accession numbers of QRT-PCR forward (FOR) and reverse (REV) primers

Gene	Sequences for primers
FGF23	FOR: CAGAGCCTATCCCAATGCCTC
	REV: GGCACTGTAGATGGTCTGATGG
GAPDH	FOR: AATGGATTTGGACGCATTGGT
	REV: TTTGCACTGGTACGTGTTGAT
miR-340-5p	FOR: GCGGTTATAAAGCAATGAGA
	REV: GTGCGTGTCGTGGAGTCG
U6	FOR: AAAGCAAATCATCGGACGACC
	REV: GTACAACACATTGTTTCCTCGGA

### Western blot

Total cellular protein was extracted from each group using radioimmunoprecipitation assay (RIPA) lysate, and protein concentration was determined using the bicinchoninic acid (BCA) method (Vazyme, Nanjing, China). Protein samples of 30 μg were processed by polyacrylamide gel electrophoresis and transferred to polyvinylidene fluoride (PVDF) membrane. 5% skim milk was blocked for 1 h at room temperature, incubated overnight with the corresponding primary antibody for FGF23 (1:1000; Abcam; cat.no.ab56326, Cambridge, MA, USA), cyclinD1 (1:1000; Servicebio; cat.no.GB11079, Wuhan, China), cyclinE1 (1:1000; Servicebio; cat.no.GB13305), CDK2 (1:1000; Cell Signaling Technology, Inc; cat.no.2546, Danvers, MA, USA), CDK6 (1:1000; Cell Signaling Technology, Inc; cat.no.3136, Danvers, MA, USA), E-Cadherin (1:1000; Abways; cat.no.AB3386, Shanghai, China) and N-Cadherin (1:1000; Abways; cat.no.CY5015, Shanghai, China), and the next day, secondary antibody was added and incubated for 1 h at room temperature for development. Protein expression was calculated by analyzing the gray values of the protein bands using ImageJ software, with GAPDH (1:1000; Servicebio; cat.no.GB12002, Wuhan, China) as an internal reference.

### Immunofluorescence

Cells were fixed with 4% paraformaldehyde for 20 min, ruptured with 0.5% TritonX-100 for 10 min, blocked with 3% fetal bovine serum albumin (BSA) for 1 h and incubated overnight at 4 °C with FGF23 antibody. Fluorescent secondary antibodies were incubated for 1 h at room temperature and stained with 4′,6-diamidino-2-phenylindole (DAPI) for 5 min. Slides were mounted with anti-fluorescence quenching mounting solution. All experiments were performed in the dark.

### Statistical analysis

Data analysis was performed by Prism Statistical software, and measurement data were expressed as mean ± standard deviation. *t* test was used for comparison between two groups, and one-way analysis of variance was used for comparison between multiple groups. *P* < 0.05 was considered statistically significant.

## Results

### FGF23 is highly expressed in osteosarcoma cells

**Fig. 1:**
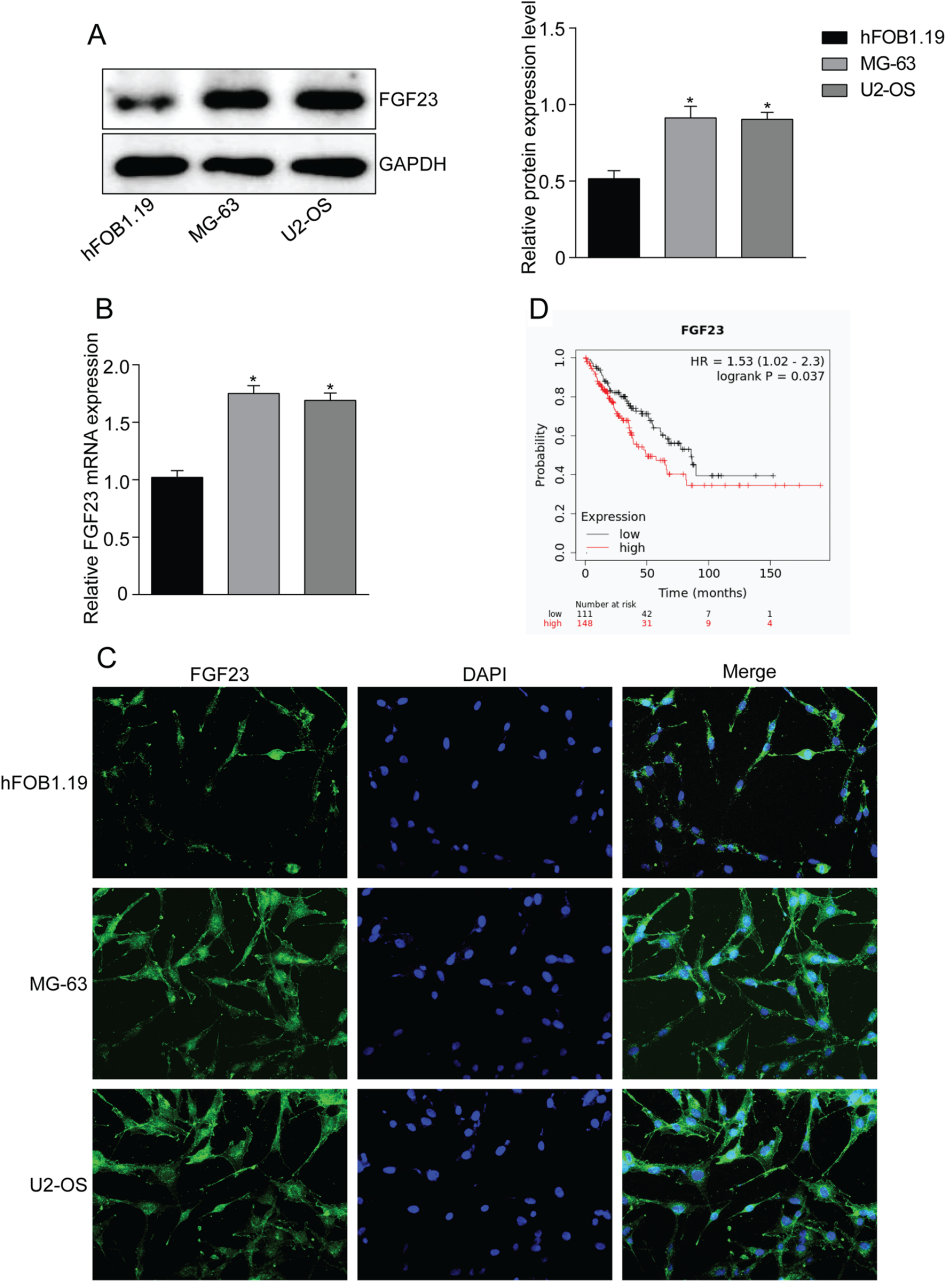
FGF23 is elevated in osteosarcoma cells and is associated with poor survival. **A** A comparative analysis of FGF23 protein expression in human osteosarcoma cells MG-63 and U2-OS versus normal human osteoblasts hFOB1.19. **B** Analysis of FGF23 mRNA expression in osteosarcoma cells. **C** Immunofluorescence staining for FGF23. **D** Kaplan–Meier curves showing that the overall survival rate of osteosarcoma is associated with high or low FGF23 expression levels. *P < 0.05 versus the hFOB1.19 group

Western blot and qRT-PCR were applied to detect protein and mRNA expression levels of FGF23 in OS cell lines (MG-63, U2-OS) and normal osteoblast cell line hFOB1.19 cells (Fig. 1A, B). The results showed that FGF23 expression was up-regulated in OS cells compared with osteoblast hFOB1.19. It also showed higher expression of FGF23 in OS cells in immunofluorescence (Fig. 1C). Moreover, the Kaplan–Meier online database analysis indicated that the overall survival rate of high FGF23 expression in OS patients was lower than that of the low FGF23 expression group (Fig. 1D). Based on the above data, we hypothesized that FGF23 is associated with OS progression.

### Overexpression of FGF23 promotes osteosarcoma cell proliferation

**Fig. 2:**
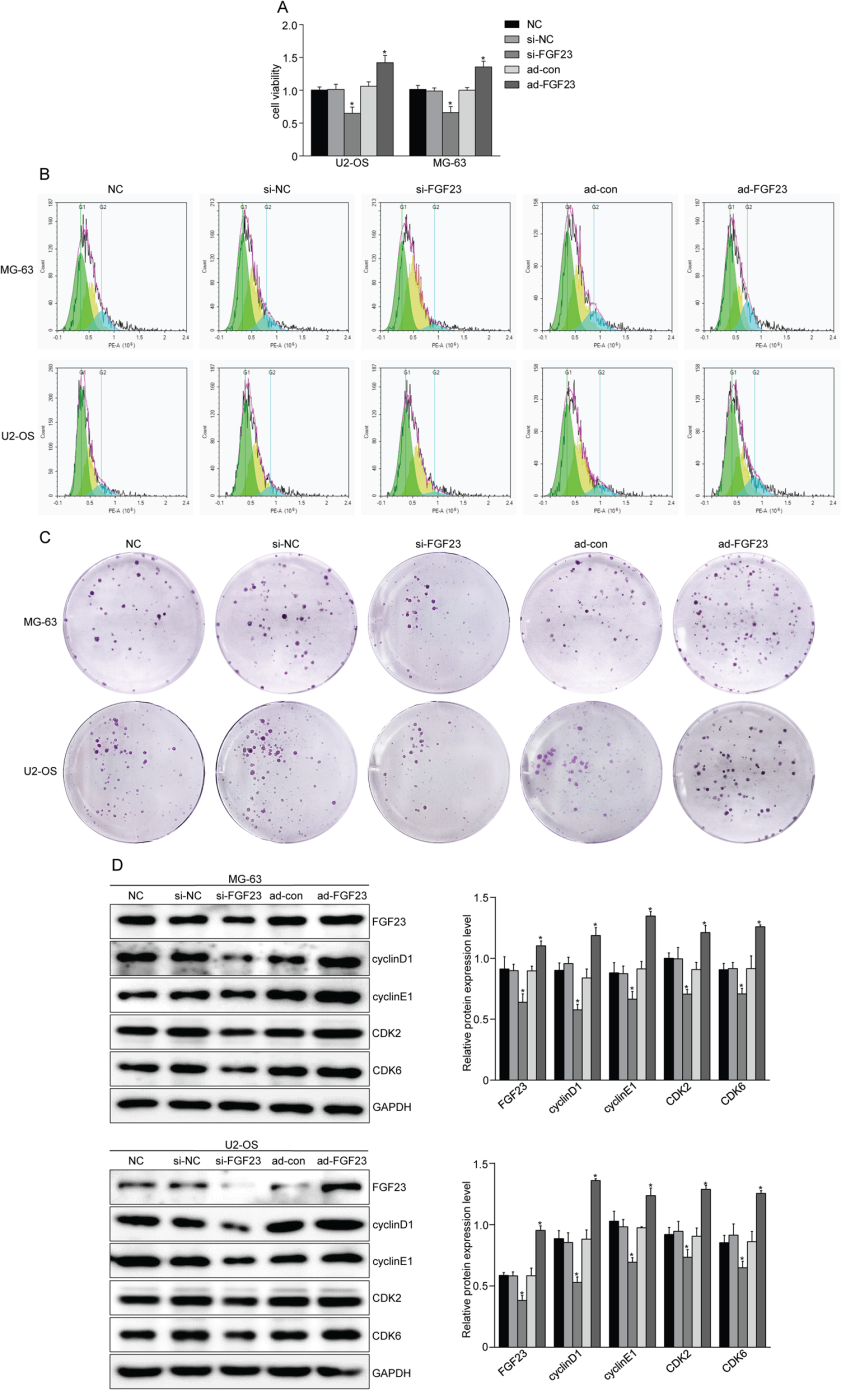
FGF23 regulates osteosarcoma cell proliferation. **A** MTT assay showed that FGF23 increased the activity of osteosarcoma cells. **B** Flow cytometry was used to detect the effect of FGF23 on the cell cycle of osteosarcoma cells. **C** Colony formation images of MG-63 and U2-OS cells stably transfected with ad-FGF23 adenovirus and si-FGF23 plasmid (14 days). **D** Western blot analysis showed that the protein levels of cell cycle-related proteins (cyclinD1, cyclinE1, CDK2, CDK6) after interfering with FGF23. *P < 0.05 versus the NC group

Adenovirus overexpressing FGF23, control adenovirus and silencing FGF23 plasmid and control plasmid were transfected into OS cells to further verify the effect of FGF23 on the development of OS. The western blot results showed that the expression of FGF23 was significantly up-regulated in the FGF23-overexpressing group compared with the control group, while FGF23 silencing significantly inhibited the expression of FGF23 (Fig. 2D). Analysis of cell viability results by both MTT and colony formation assays indicated that cell viability was significantly higher in the FGF23-overexpressing group compared with the NC group (*P* < 0.05) (Fig. 2A). Flow cytometry analysis of cell cycle results showed that the proportion of cells in G0/G1 phase decreased, while the proportion of cells in S and G2/M phases significantly increased in the overexpression FGF23 group (Fig. 2B). FGF23 overexpression promotes colony formation ability of OS cells (Fig. 2C). Moreover, CDK2, CDK6, cyclinD1 and cyclinE1, proteins associated with proliferation, were up-regulated in the ad-FGF23 group. However, silencing of FGF23 in OS cells partially reversed the above results (Fig. 2D). These data suggest that FGF23 has the ability to promote osteosarcoma cell proliferation.

### FGF23 promotes the migration and invasion of osteosarcoma cells

**Fig. 3:**
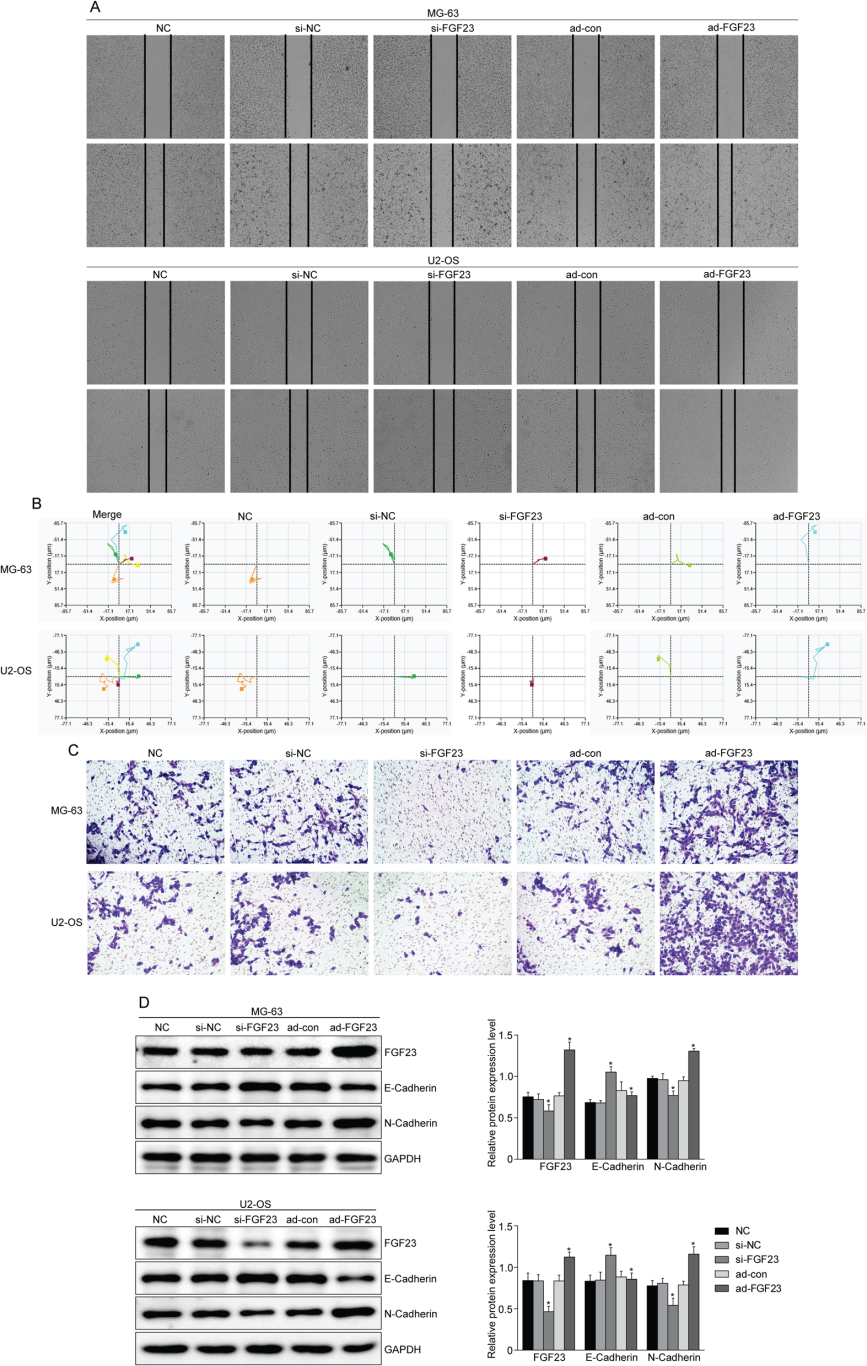
FGF23 promotes osteosarcoma cell migration and invasion. **A** Scratch assay was used to detect the effect of interfering with FGF23 expression on osteosarcoma cell migration. **B** Holomonitor ® M4 was used to track the trajectory of single cell motility. **C** Transwell cell invasion assay was used to detect the effect of interfering with FGF23 expression on osteosarcoma cell invasion. **D** Western blot was used to analyze the protein expression levels of EMT-related proteins (E-Cadherin, N-Cadherin) after interfering with FGF23

The effect of interfering with FGF23 expression on the migration of OS cells was studied by scratch assay (Fig. 3A). The results showed that the cell migration ability of ad-FGF23 group was significantly higher than that of NC group. Moreover, individual cells were followed up by a holographic cell imaging analyzer, and the migration distance of cells in the ad-FGF23 group was found to be significantly increased compared with the control group (Fig. 3B). The results of transwell cell invasion assay showed that the number of penetrating cells in the FGF23-overexpressing group was significantly more than that in the NC group (Fig. 3C). In addition, Western blot results showed that N-Cadherin expression levels were up-regulated in the ad-FGF23 group, while E-Cadherin expression was down-regulated (Fig. 3D). From this part of the results, it can be found that FGF23 can promote osteosarcoma cell migration, invasion and epithelial-mesenchymal transition (EMT).

### FGF23 targets miR-340-5p

**Fig. 4:**
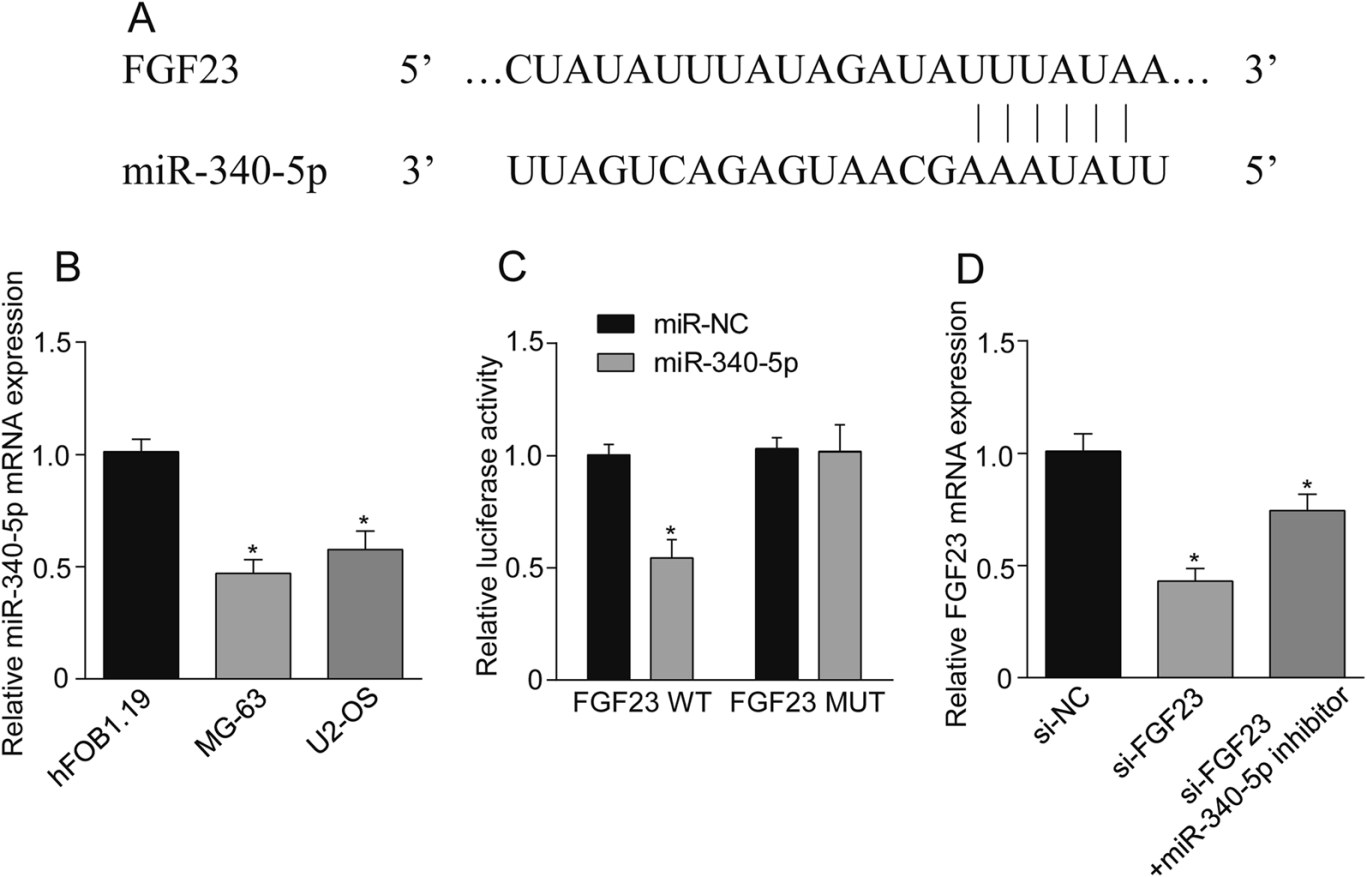
FGF23 targets miR-340-5p. **A** TargetScan online database analysis predicted the presence of binding sites between FGF23 and miR-340-5p. **B** QRT-PCR was used to detect the expression level of miR-340-5p in osteosarcoma cells. **C** Dual-luciferase gene reporter assay showed that miR-340-5p mimic inhibited the luciferase activity of FGF23-WT. **D** The effect of miR-340-5p inhibitor on FGF23 expression

**Fig. 5:**
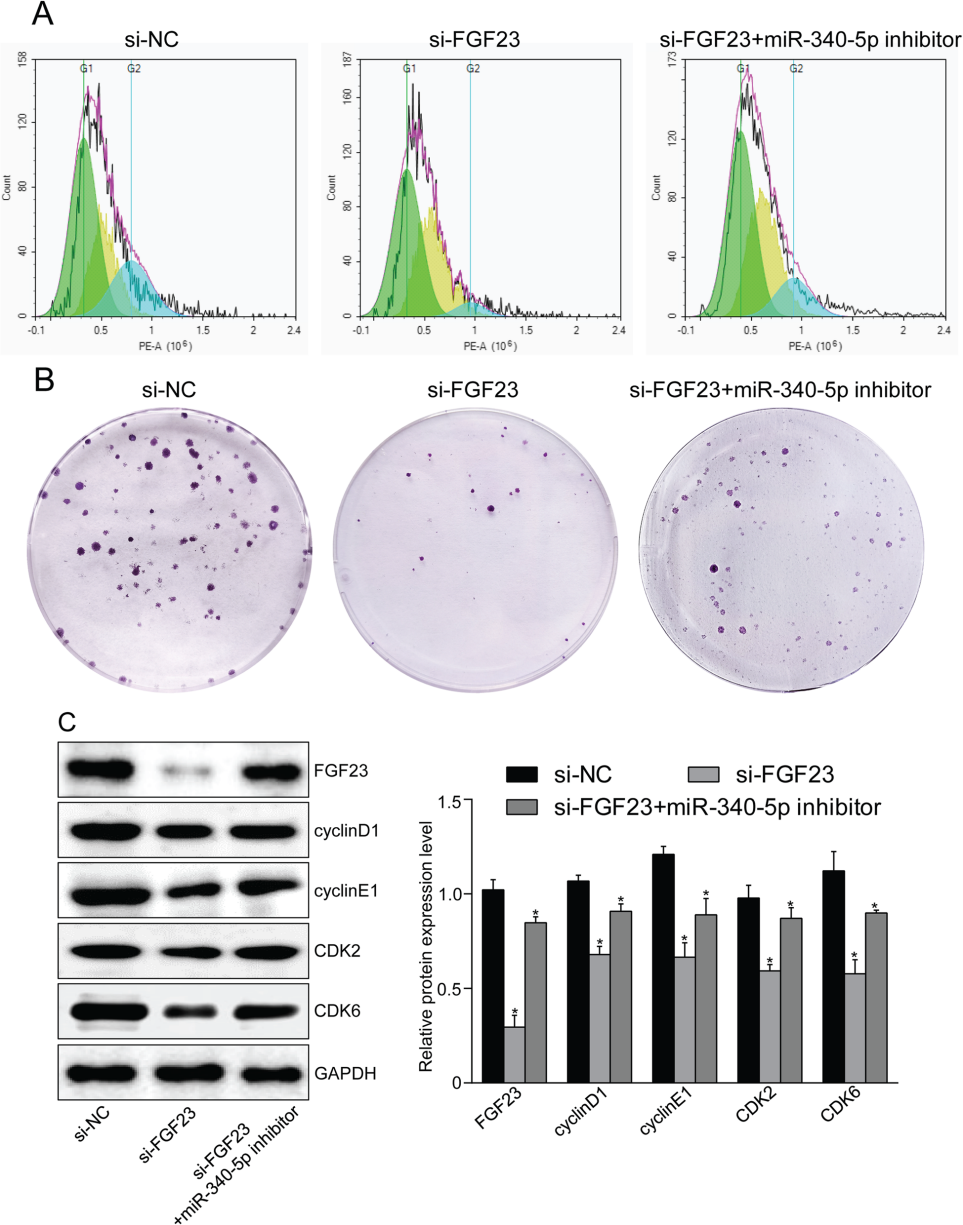
FGF23 targets miR-340-5p to promote osteosarcoma cell proliferation. **A** Cell cycle by flow cytometry. **B** Colony formation assay showed accelerated cell proliferation after transfection with miR-340-5p inhibitor. **C** Protein levels of cell cycle-related proteins (cyclinD1, cyclinE1, CDK2, CDK6) after miR-340-5p inhibition

Targets predicted to potentially bind to FGF23 by TargetScan, an online database, revealed partial specific binding sites between miR-340-5p and FGF23 (Fig. 4A). QRT-PCR detected the expression of miR-340-5p OS cells and found that miR-340-5p was significantly down-regulated in OS cells compared with human osteoblasts hFOB1.19 (Fig. 4B). Luciferase analysis report showed that FGF23 wild-type luciferase activity in miR-340-5p group was lower than that in miR-NC group (*P* < 0.05), and there was no significant difference in FGF23 mutant luciferase between miR-340-5p group and miR-NC group (*P* > 0.05) (Fig. 4C). Moreover, Western blot and qRT-PCR results revealed that interference with miR-340-5p affected FGF23 expression, which was up-regulated after transfection with miR-340-5p inhibitor (Figs. 4D, 5C). It can be seen that FGF23 negatively regulates miR-340-5p.

### The effect of FGF23 on osteosarcoma is regulated by miR-340-5p

**Fig. 6:**
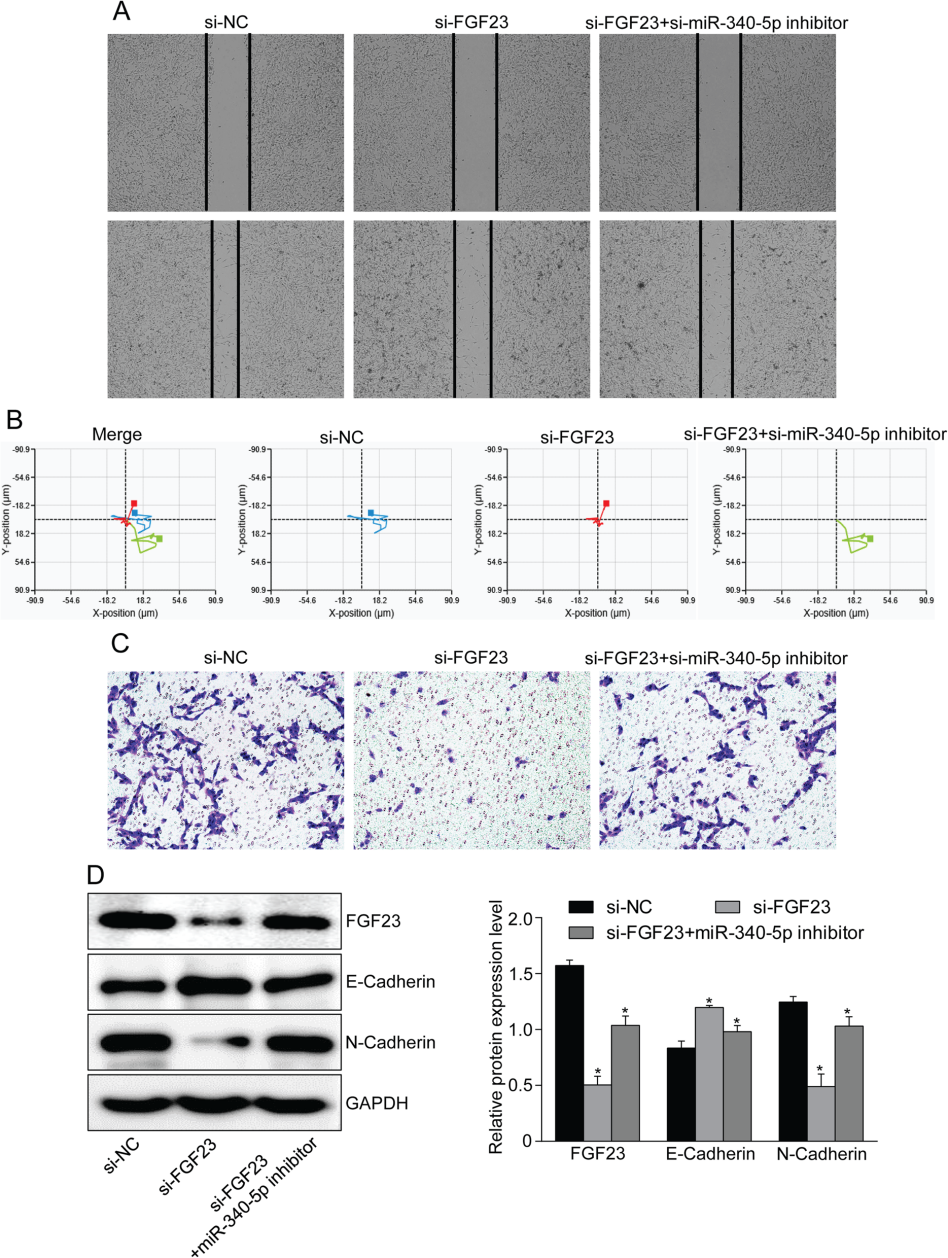
FGF23 promotes osteosarcoma cell migration and invasion by inhibiting miR-340-5p. **A**, **B** Scratch assay and holographic cell imaging showed that miR-340-5p inhibitor could reverse the decreased migration ability caused by FGF23 silencing. **C** Transwell cell invasion assay showed that miR-340-5p inhibition promoted MG-63 cell invasion. **D** Protein expression levels of EMT-related proteins (E-Cadherin, N-Cadherin) transfected with miR-340-5p inhibitor

To verify whether FGF23 affects proliferation and migration of OS cells by targeting miR-340-5p, we transfected the miR-340-5p inhibitor into MG-63 cells that had been transfected with the si-FGF23 plasmid. FGF23 knockdown could inhibit the proliferation ability of OS cells, while transfection with miR-340-5p inhibitor partially reversed this result (Fig. 5). Moreover, co-transfection of si-FGF23 and miR-340-5p inhibitor reduced the inhibitory effect of si-FGF23 on MG-63 cell migration and invasion. In addition, according to protein changes associated with proliferation and EMT progression, the promotion of OS proliferation and EMT progression after overexpression of FGF23 was reversed after transfection with miR-340-5p inhibitor (Fig. 6).

## Discussion

Osteosarcoma is a primary malignant bone tumor with high metastasis and high recurrence, for which there is no effective treatment. In this study, we examined the expression of FGF23 in OS cells and found that FGF23 was highly expressed, and that its high expression was associated with a lower overall survival rate of OS patients. In addition, silencing FGF23 inhibited the proliferation, migration and invasion of OS cells in vitro.

Firstly, we found that OS patients with high FGF23 expression had a lower overall survival rate than those with low expression by Kaplan–Meier online database analysis. So, we hypothesized that FGF23 may affect the prognosis of OS patients by regulating OS cell proliferation, migration and invasion. FGF23 is a bone-derived sex hormone that regulates the metabolism of phosphorus and active vitamin D [5]. FGF23 can act on parathyroid gland and kidney through MAPK/ERK signaling pathway to regulate the interaction such as calcium, phosphorus and other minerals, vitamin D and parathyroid hormone, and indirectly regulate the body's bone metabolism [17, 18]. Tumor patients with high FGF23 expression who develop calcium and phosphorus metabolism disorders is more likely to exacerbate the deterioration of the disease. FGF23 gene expression has been found to be tightly associated with the development of prostate cancer, and FGF23 predicts a worse prognosis in prostate cancer [19]. FGF23 is highly expressed in prostate and colorectal cancer, and colorectal cancer with high FGF23 expression is prone to calcium and phosphorus metabolism disorders [20]. In the present study, we showed that FGF23 expression was elevated in OS cells. And FGF23 can promote the proliferation of OS cells and improve the motility and invasiveness of cells. These data indicate that FGF23 plays an important role in the development and progression of OS, so it may be used as an effective target in the diagnosis and targeted therapy of OS.

To further identify the molecular mechanism by which FGF23 acts on OS cells, we predicted FGF23 and miR-340-5p binding by TargetScan. Targeting of FGF23 and miR-340-5p was verified with a dual-luciferase reporter assay. And miR-340-5p inhibitor could promote the expression level of FGF23 in OS cells by western blot. Several studies have shown that miR-340-5p is involved in the development and progression of a variety of tumors. The expression of miR-340-5p decreased in colon cancer tissues and overexpression of miR-340-5p significantly inhibited the proliferative activity as well as invasive ability of tumor cells [11]. Up-regulation of miR-340-5p expression in lung adenocarcinoma could significantly down-regulate the invasion and metastasis ability of tumor cells and enhance the antitumor activity of the drug [21]. In endometrial cancer, miR-340-5p inhibits epithelial-mesenchymal transition in cancer cells [12]. These results all suggest that miR-340-5p plays an important role in the development and progression of human cancer. In addition, low expression level of miR-340-5p in OS cells could inhibit the progression of OS by targeting Wnt/β-catenin the negative regulatory signaling pathway of STAT3 gene [22]. RUSC1-AS1 promotes osteosarcoma development in vitro and in vivo by adsorbing miR-340-5p and activating the PI3K/AKT signaling pathway [13]. These results are consistent with our findings. In this study, we observed that miR-340-5p was down-regulated in OS cells and negatively correlated with FGF23 expression. MiR-340-5p inhibitor partially reversed the inhibitory effect of si-FGF23 on the proliferation, migration and invasion of osteosarcoma cells. FGF23 may therefore affect OS cell proliferation, migration, invasion and EMT progression by regulating miR-340-5p.

## Conclusion

FGF23 is highly expressed in osteosarcoma cells and can promote the ability of osteosarcoma cells to proliferate and migrate. MiR-340-5p is identified as a direct target gene of FGF23. FGF23 can target miR-340-5p to regulate the development and progression of osteosarcoma cells. The results of this study suggest that FGF23 may be an effective molecular target for the clinical treatment of osteosarcoma.

## Data Availability

The datasets generated and analyzed during the present study are available from the corresponding author on reasonable request.
